# 313. Performance of Rapid Diagnostic Testing at Days 4-6 from Diagnosis: Implications for Discharge from Isolation on a University Campus

**DOI:** 10.1093/ofid/ofac492.391

**Published:** 2022-12-15

**Authors:** Joseph Atarere, Zhenwei Zhou, Jacquelyn Turcinovic, Scott Seitz, Cole Sher-Jan, Madison Gilbert, Laura F White, Mohammad Hossain, Victoria Overbeck, Roxanne M Mistry, Lynn Doucette-Stamm, Judy T Platt, Hannah E Nichols, Davidson H Hamer, Catherine M Klapperich, Karen R Jacobson, John Connor, Tara C Bouton

**Affiliations:** Boston Medical Center/ MedStar Health, Baltimore, Maryland; Boston University, Boston, Massachusetts; Boston University, Boston, Massachusetts; Boston University, Boston, Massachusetts; BUMC, Somerville, Massachusetts; Boston Medical Center, Boston, Massachusetts; Boston University School of Public Health, Boston, Massachusetts; Boston University, Boston, Massachusetts; Boston University School of Public Health, Boston, Massachusetts; Boston Medical Center, Boston, Massachusetts; Boston University, Boston, Massachusetts; Boston Iniversity, Boston, Massachusetts; Boston University, Boston, Massachusetts; Boston University School of Public Health, Boston, Massachusetts; Boston University College of Engineering, Boston, Massachusetts; Boston University School of Medicine, Boston, Massachusetts; Boston University, Boston, Massachusetts; Boston Medical Center and Boston University School of Medicine, Boston, Massachusetts

## Abstract

**Background:**

Omicron rapidly replaced delta as the predominant strain causing COVID-19 related illness in the United States (US) in December 2021, the same month the US CDC reduced the recommended isolation period from 10 to 5 days for asymptomatic individuals or those with resolving symptoms. New evidence suggests some asymptomatic individuals with omicron remain culture positive beyond 5 days from diagnosis. We sought to evaluate the performance of a SARS-CoV-2 antigen rapid diagnostic test (RDT) in predicting persistent potential for transmission at the end of a five-day isolation period among young, fully vaccinated individuals in a university community setting.

**Methods:**

A subgroup of participants enrolled in a longitudinal COVID-19 cohort were asked to self-perform RDTs on days 4 to 6 from diagnostic test date in addition to a separate self-collected anterior nasal swab used for culture and RT-PCR, and a daily symptom screen (15 COVID-19 symptom questions on a 4-point scale). We calculated the daily and overall sensitivity and specificity of the RDTs in comparison to SARS-CoV-2 culture result. We also compared the N1 cycle threshold (CT) values and symptom score on each day of the study by RDT results.

**Results:**

Of 23 participants, the mean age was 20 years, all had completed their primary COVID-19 vaccine series, and 13 (65.0%) had received a booster vaccine (Table 1). Compared to culture, sensitivity and specificity of the RDTs were 100% and 62% respectively (Table 2). Compared to participants with negative RDTs, median CT values were lower in those with positive RDTs on each day of the study (Figure 1). Participants who had positive RDTs on all three days had higher symptom scores (Figure 2) than those without.

**Figure:**
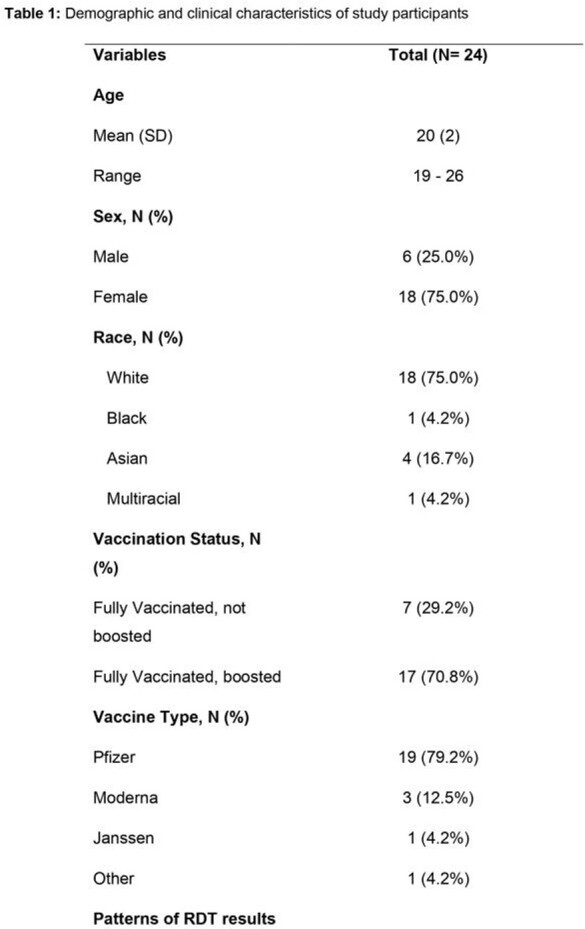

**Figure:**
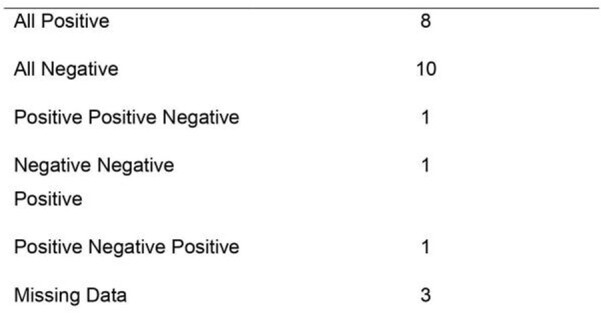

**Figure:**
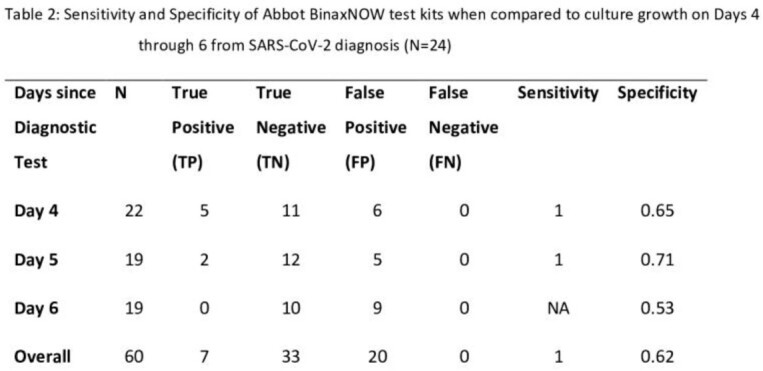

**Conclusion:**

RDTs have a high sensitivity in detecting culture positive SARS-CoV-2 on Days 4 to 6 from initial diagnostic test. However, the high false positive rate of 38% means that over a third of culture negative individuals will stay in isolation longer than necessary if RDTs are used in test to release from isolation protocols. Viral loads (CT values) and symptom scores were higher for participants with persistently positive RDT result. An approach that uses a combination of RDTs, CT values and symptom score may prove useful in guiding isolation duration.

**Figure:**
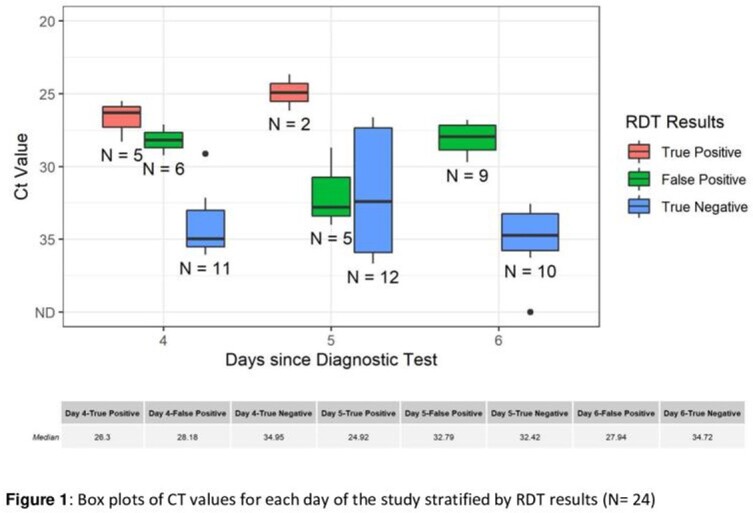

**Figure:**
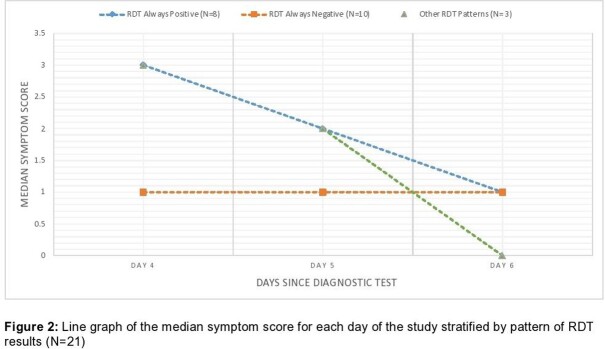

**Disclosures:**

**Davidson H. Hamer, MD**, Trinity Biotech: Advisor/Consultant **Catherine M. Klapperich, Ph.D.**, BioSens8, LLC: Ownership Interest.

